# Molecular response to the pathogen *Phytophthora sojae* among ten soybean near isogenic lines revealed by comparative transcriptomics

**DOI:** 10.1186/1471-2164-15-18

**Published:** 2014-01-10

**Authors:** Feng Lin, Meixia Zhao, Douglas D Baumann, Jieqing Ping, Lianjun Sun, Yunfeng Liu, Biao Zhang, Zongxiang Tang, Elisa Hughes, Rebecca W Doerge, Teresa J Hughes, Jianxin Ma

**Affiliations:** 1 Department of Agronomy, Purdue University, West Lafayette, IN 47907, USA; 2 Department of Mathematics, University of Wisconsin – La Crosse, La Crosse, WI 54601, USA; 3 Department of Statistics, Purdue University, West Lafayette, IN 47907, USA; 4 Department of Botany and Plant Pathology, Purdue University, West Lafayette, IN 47907, USA; 5 USDA-ARS Crop Production and Pest Control Research Unit, Purdue University, West Lafayette, IN 47907, USA

**Keywords:** Comparative transcriptomics, Resistance to *Phytophthora sojae*, Soybean

## Abstract

**Background:**

Phytophthora root and stem rot (PRR) of soybean, caused by *Phytophthora sojae*, is controlled by *Rps* genes. However, little is known regarding the *Rps*-induced molecular responses to *P. sojae* and how they actually overlap. We thus sequenced, analyzed, and compared the transcriptomes of 10 near isogenic lines (NILs), each with a unique *Rps* gene/allele, and the susceptible parent Williams, pre- and post-inoculation with the pathogen.

**Results:**

A total of 4,330 differentially expressed genes (DEGs) were identified in Williams versus 2,014 to 5,499 DEGs in individual NILs upon inoculation with the pathogen. Comparisons of the DEGs between the NILs and Williams identified incompatible interaction genes (IIGs) and compatible interaction genes (CIGs). Hierarchical cluster and heatmap analyses consistently grouped the NILs into three clusters: Cluster I (*Rps1-a*), Cluster II (*Rps1-b*, *1-c* and *1-k*) and Cluster III (*Rps3-a*, *3-b*, *3-c*, *4*, *5*, and *6*), suggesting an overlap in *Rps*-induced defense signaling among certain NILs. Gene ontology (GO) analysis revealed associations between members of the WRKY family and incompatible reactions and between a number of phytohormone signaling pathways and incompatible/compatible interactions. These associations appear to be distinguished according to the NIL clusters.

**Conclusions:**

This study characterized genes and multiple branches of putative regulatory networks associated with resistance to *P. sojae* in ten soybean NILs, and depicted functional “fingerprints” of individual *Rps*-mediated resistance responses through comparative transcriptomic analysis. Of particular interest are dramatic variations of detected DEGs, putatively involved in ethylene (ET)-, jasmonic acid (JA)-, (reactive oxygen species) ROS-, and (MAP-kinase) MAPK- signaling, among these soybean NILs, implicating their important roles of these signaling in differentiating molecular defense responses. We hypothesize that different timing and robustness in defense signaling to the same pathogen may be largely responsible for such variations.

## Background

Phytophthora root and stem rot (PRR) is one of the most devastating diseases of soybean (*Glycine max*), causing nearly $200 million in annual yield losses in the U.S. alone [1]. PRR is caused by the soil-borne, hemibiotrophic pathogen *Phytophthora sojae*, and is most effectively controlled by *Rps* (Resistance to *P. sojae*) genes. The resistance conferred by an *Rps* gene is race specific, and the interaction between an *Rps* gene and the corresponding avirulence (*Avr*) gene in *P. sojae*, follows the gene-for-gene model [2]. Currently, 18 *Rps* genes/alleles from soybean and 12 *Avr* genes from *P. sojae* have been identified [3-5].

Like most resistance (*R*) genes, the *Rps* gene family encodes, or is predicted to encode nucleotide binding leucine rich repeat (NB-LRR)-type proteins [6-8], which are able to recognize the *Avr* effector proteins of pathogens directly or indirectly to induce the appropriate defense response [9,10]. The first evidence of a direct interaction between an *R* and *Avr* protein was reported in the flax-*Melampsora lini* pathosystem [11]. Indirect interactions, however, are more common. In these cases, the *R* protein appears to require the existence of a ‘guard protein’ or ‘decoy’ in order to recognize an *Avr* protein [12,13]. A classical example of this type of interaction is the RPM1-interacting protein 4 (RIN4), which is ‘guarded’ by RPM1 (RESISTANCE TO PSEUDOMONAS SYRINGAE PV MAC*ULICOLA 1)* and RPS2 (RESISTANCE TO *P. SYRINGAE* 2) proteins in *Arabidopsis*[14,15].

The recognition of pathogen effectors triggers an innate immunity response that is mediated by two distinct types of NB-LRR proteins, the toll-interleukin-1 receptor (TIR)-NB-LRR proteins and the coiled-coil (CC)-NB-LRR proteins. The former requires EDS1 (ENHANCED DISEASE SUSCEPTIBILITY 1), a central regulator of effector-triggered immunity (ETI), which functions together with PAD4 (PHYTOALEXIN DEFICIENT 4). In contrast, the latter NB-LRR proteins are independent of EDS1 but require NDR1 (NON-RACE-SPECIFIC DISEASE RESISTANCE 1) [16-21]. The interaction among these intracellular proteins results in a regulation network of phytohormone signaling, which is mainly mediated by salicylic acid (SA) for biotrophic and hemibiotrophic pathogens, and jasmonic acid (JA) and ethylene (ET) for necrotrophic pathogens [8,22]. In addition to SA, JA and ET, other phytohormones such as brassinosteroids (BR), abscisic acid (ABA), auxin, gibberellins (GA) and cytokinin (CK) also contribute to plant immune responses, with complex crosstalk between one another [23].

It is suggested that the resistance to *P. sojae* conferred by *Rps* genes is mediated by the SA signaling pathway, with the induction of SA-mediated systemic acquired resistance (SAR) occurring several days post infection (dpi) via expression of the gene *NPR1*[24-26]. During this process, two putative regulators of the chromosome condensation 1 (*RCC1*) gene family are down-regulated during the incompatible interaction [27]. Besides SA, exogenous treatment of 1-aminocyclopropane-1-carboxylic acid (ACC, a precursor of ET) has been shown to enhance resistance while applications of GA and ABA induce susceptibility, highlighting the complexity of phytohormone signaling pathways in response to attack by *P. sojae*[26,28,29].

A microarray study of transcriptomes from one susceptible and two partially resistant soybean genotypes indicated that 97-99% of all detectable genes experienced transcriptional modulation five dpi in response to infection by *P. sojae*[30]*.* However, the majority of these differences were less than two fold. Another microarray study of transcriptomic changes in soybean revealed a peak in most defense related genes at 24 hours post inoculation (hpi) with *P. sojae*[24]. Recently, Zhang et al conducted a proteomics study in which 46 differentially expressed proteins were identified in soybean after infection with *P. sojae*[31]. Among these, 26 were affected during the incompatible interaction, while the other 20 were altered during the compatible interaction. These studies have contributed to our understanding of the interaction between soybean and *P. sojae*. Nevertheless, what mechanisms underlie compatible and incompatible interactions, how molecular responses mediated by a variety of *Rps* genes/alleles resemble or differ from one another, as well as the nature of these similarity or difference, remain largely unknown.

Access to the soybean genome sequence of Williams 82 (*Rps1-k*) [32], and the advent of high-throughput RNA sequencing (RNA-Seq) by next-generation sequencing technology, have allowed researchers to take a novel look at the molecular interaction between soybean and *P. sojae*. Kim et al reported the first application of RNA-Seq for profiling gene expression in soybean in response to pathogen attack [33]. In this study, the transcriptomes of two near isogenic lines (NILs), one resistant and one susceptible to bacterial leaf pustule, were analyzed 0, 6, and 12 hpi and a total of 2,761 differentially expressed genes (DEGs), including a set of defense response genes, were identified.

NILs are pure breeding lines that are developed by backcrossing a donor line with the recurrent parent for at least five generations to achieve introgression of a desired trait. As such, they share more than 98% genetic identity with the recurrent parent, except for the region where the desired gene is located. In order to determine the molecular mechanisms responsible for *Rps*-mediated defense and to understand molecular basis for diverse responses to the pathogen, we analyzed the transcriptomes of 10 NILs, each with a unique *Rps* gene/allele, along with the susceptible recurrent parent Williams, pre- and post-inoculation with a race 1 isolate of *P. sojae* (avirulent towards NILs; virulent towards Williams).

## Results

### Symptom development in 10 NILs and the recurrent susceptible parent inoculated with *P. sojae*

The 10 soybean NILs carrying *Rps1-a*, *Rps1-b*, *Rps1-c*, *Rps1-k*, *Rps3-a*, *Rps3-b*, *Rps3-c, Rps4*, *Rps5*, or *Rps6*, within the Williams background, provided a unique resource for investigating the common and specific defense responses mediated by individual *Rps* genes against *P. sojae* (Additional file 1). Phenotypic reactions of the 10 NILs and Williams to *P. sojae* 7 days post-inoculation (dpi) with the pathogen were evaluated under greenhouse conditions. In each of three experimental replications, approximately half the number of seedlings from each line was inoculated with race 1 of *P. sojae.* The remaining seedlings were “mock” inoculated in the same manner without the pathogen. In each experimental replicate, Williams was susceptible (expanding lesion/plant death) to *P. sojae*, while all NILs were resistant (Additional file 2). NILs containing *Rps1-a*, *Rps1-b*, *Rps1-c*, *Rps1-k *and *Rps4* displayed 100% survival when challenged with the pathogen. In contrast, NILs containing *Rps3-a*, *Rps3-b*, *Rps3-c*, *Rps5* and *Rps6* showed a slight variation in the proportion of surviving seedlings across the three replicates, which may be attributed to minor differences in environmental conditions. Despite this variation, the percentage of surviving seedlings in each replicate was equal to or greater than 75%, which is generally used as a criterion for defining a pure line resistance [34]. All mock-inoculated seedlings were asymptomatic of PRR (Additional file 2).

### Transcriptional changes in 10 NILs and the recurrent susceptible parent in response to *P. sojae*

RNAs, representing the entire transcriptomes of *P. sojae* inoculated and mock-inoculated seedlings from each of the 10 NILs and Williams 24 hpi with the pathogen, were sequenced and 14.5 to 50.1 million raw reads were generated for individual samples (Table 1). Trimming adaptor sequences and elimination of low quality reads and reads shorter than 101 bp, resulted in 13.5 to 39.5 million clean reads for each sample. Among these, 71.5% to 87.8% per sample were uniquely mapped to the soybean reference genome v1.0 (Table 1).

**Table 1:** Statistics of pair-end reads in RNA sequencing experiment

**Soybean line**	**Raw reads**	**Clean reads**^**a**^	**Uniquely mapped reads**^**b**^	**% of uniquely mapped reads**^**c**^
**Inoculated Group**				
Williams (*rps*)	50,126,404	39,475,154	28,222,946	71.5
Union (*Rps1-a*)	25,614,448	20,169,804	14,969,954	74.2
L77-1863 (*Rps1-b*)	28,308,350	22,460,578	16,591,206	73.9
L75-3735 (*Rps1-c*)	19,696,090	15,483,052	11,084,896	71.6
Williams82 (*Rps1-k*)	14,508,392	13,527,604	11,527,676	85.2
L83-570 (*Rps3-a*)	21,982,116	17,531,234	12,954,567	73.9
L91-8347 (*Rps3-b*)	32,063,808	25,210,242	18,704,692	74.2
L92-7857 (*Rps3-c*)	29,003,280	22,775,218	16,776,075	73.7
L85-2352 (*Rps4*)	27,891,876	21,857,446	15,860,686	72.6
L85-3059 (*Rps5*)	35,204,260	30,651,204	24,816,154	81.0
L89-1581 (*Rps6*)	26,761,058	23,224,142	19,322,975	83.2
**Mock-inoculated Group**				
Williams (*rps*)	21,949,578	20,385,960	17,841,238	87.5
Union (*Rps1-a*)	25,680,502	23,860,934	20,936,764	87.7
L77-1863 (*Rps1-b*)	20,303,214	18,830,218	16,453,627	87.4
L75-3735 (*Rps1-c*)	26,452,482	22,841,526	19,049,873	83.4
Williams82 (*Rps1-k*)	19,289,852	16,845,090	14,241,574	84.5
L83-570 (*Rps3-a*)	33,576,796	29,006,366	24,493,712	84.4
L91-8347 (*Rps3-b*)	25,609,764	23,775,078	20,880,124	87.8
L92-7857 (*Rps3-c*)	24,806,160	22,983,728	20,078,725	87.4
L85-2352 (*Rps4*)	24,677,426	22,899,582	20,085,744	87.7
L85-3059 (*Rps5*)	26,874,804	23,304,436	19,637,730	84.3
L89-1581 (*Rps6*)	27,363,314	25,362,532	22,151,262	87.3

^a^Reads with a quality score less than 20 and length less than 101 nucleotides were excluded.

^b^Reads uniquely mapped to *G. max* genome assembly (v1.0) using TopHat 2.0 software [58].

^c^Percentage of uniquely mapped reads out of clean reads.

Based on the 46,367 high-confidence gene models annotated in the soybean reference genome [32], the relative change in the abundance of reads mapped to the same genes for inoculated and mock-treated samples, and the criteria for defining DEGs pre- and post-inoculation, a total of 9,847 non-redundant gene models were identified as DEGs in at least one of the 11 soybean lines. The numbers of DEGs identified between Williams and the 10 NILs or among the 10 NILs vary greatly. For example, 2,014 to 2,366 DEGs were found in individual NILs containing *Rps1-a*, *Rps1-b*, *Rps1-c,* or *Rps1-k*, while 3,038 to 5,499 DEGs were detected in individual NILs containing *Rps3-a*, *Rps3-b*, *Rps3-c*, *Rps4*, *Rps5* or *Rps6* (Table 2). Among the 10 NILs, 1,274 to 2,823 DEGs were up-regulated, while 643 to 2,744 DEGs were down-regulated in response to inoculation with the pathogen (Table 2). In comparison, 2,460 up-regulated and 1,870 down-regulated DEGs were identified in Williams upon inoculation with *P. sojae*.

**Table 2:** Differentially expressed genes (DEGs) in each soybean line

**Soybean line**	**Up-regulated DEGs**	**Down-regulated DEGs**	**Total**
Williams (*rps*)	2460	1870	4330
Union (*Rps1-a*)	1432	643	2075
L77-1863 (*Rps1-b*)	1536	831	2366
L75-3735 (*Rps1-c*)	1532	698	2230
Williams82 (*Rps1-k*)	1274	740	2014
L83-570 (*Rps3-a*)	2362	1827	4189
L91-8347 (*Rps3-b*)	2823	2166	4989
L92-7857 (*Rps3-c*)	2749	2247	4996
L85-2352 (*Rps4*)	2470	2143	4613
L85-3059 (*Rps5*)	2755	2744	5499
L89-1581 (*Rps6*)	2033	1005	3038

To validate DEGs profiled by RNA-Seq analysis, six detected DEGs per soybean line (Additional file 3) were randomly chosen for quantitative RT-PCR (qRT-PCR) analysis with the same RNA samples as used for RNA-Seq. Subsequently, the patterns of differential expression of these genes detected by RNA-Seq and qRT-PCR were compared. As shown in Additional file 4, significant correlations between the patterns of DEGs detected by the two approaches were observed for each set of genes chosen from 11 individual lines (Pearson’s correlation coefficient (r), ranging from 0.725 to 0.994), indicating the reliability of DEGs identified by RNA-Seq (Additional file 4).

### Characterization of genes associated with incompatible, compatible, and basal interactions

**Figure 1:**
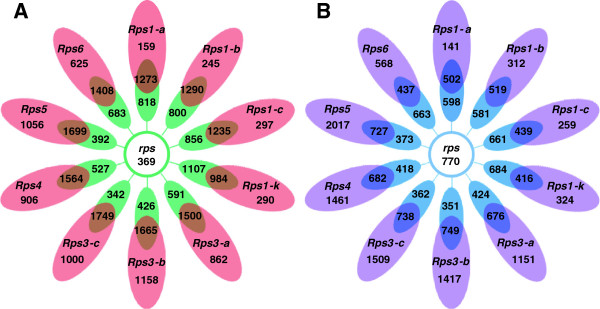
**Comparison of differentially expressed genes (DEGs) between Williams (susceptible to *Phytophthora sojae*) and 10 near isogenic lines (NILs) each containing a single *Rps *(Resistance to *P*. *sojae*) gene. A**: up-regulated DEGs. **B**: down-regulated DEGs. Red and purple represent the number of DEGs specific to individual *Rps* genes and collectively referred to as incompatible interaction genes. Brown and dark purple represent the number of DEGs shared by an individual NIL and Williams. Green and light blue represent the number of DEGs specifically expressed in ‘Williams’ when compared to a specific NIL. The central green and light blue circles represent the common proportion of Williams specific DEGs that are collectively referred to as compatible interaction genes.

In an attempt to decipher the molecular basis of resistance and susceptibility to *P. sojae*, we applied a comparative transcriptomics approach to identify DEGs specifically associated with a host response. Three major groups of genes were classified: 1) Incompatible interaction genes (IIGs), are DEGs identified in NILs and associated with resistance; 2) Compatible interaction genes (CIGs) are DEGs identified in Williams and specifically associated with the susceptible response; and 3) Basal interaction genes (BIGs), which are DEGs shared by all NILs and Williams (Figure 1).

A total of 5,806 non-redundant IIGs, 1139 CIGs, and 835 BIGs were identified (Figure 1). The number of up-regulated and down-regulated IIGs range from 159 to 1,158 and from 141 to 2,017, respectively, among the 10 NILs. Of the 1,139 CIGs, 369 were up-regulated and 770 were down-regulated; and of the 835 BIGs, 696 were up-regulated and 139 were down-regulated (Figure 1).

### Clusters of *Rps*-mediated IIGs

**Figure 2:**
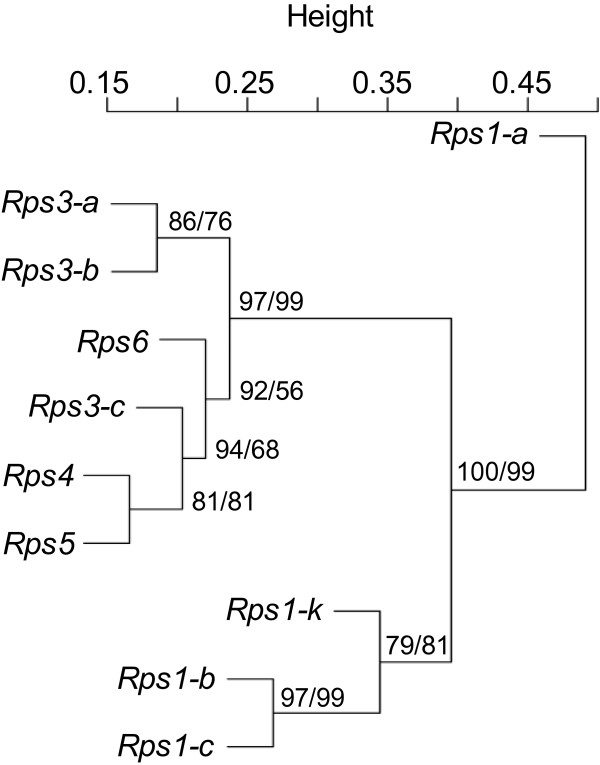
**Hierarchical cluster analysis (pvclust) of incompatible interaction genes identified for 10 soybean near isogenic lines, each containing a single *Rps *gene.** The numbers behind each node represent AU (Approximately Unbiased) / BP (Bootstrap Probability) for an estimation of the confidence of each node.

To understand how transcriptomic changes mediated by different *Rps* genes/alleles overlap, we performed hierarchical cluster analysis using pvclust, an R package for assessing the uncertainty in hierarchical clustering [35], with log_2_ fold change (FC) of the 5,806 IIGs identified in at least one of the ten NILs. The NILs were grouped into three clusters, Clusters I, II, and III (Figure 2). Cluster I (C-I) consisted of the NIL containing *Rps1-a* only. Cluster II (C-II) was composed of those NILs containing *Rps1-b*, *Rps1-c* and *Rps1-k*, while Cluster III (C-III) included those NILs containing *Rps3-a*, *Rps3-b*, *Rps3-c*, *Rps4*, *Rps5*, and *Rps6.* This clustering of IIGs was further supported by the hierarchical cluster structure of the ten NILs revealed by heatmap analysis [36] (Additional file 5). In addition, the numbers of IIGs identified for each individual NIL, were unevenly distributed among different clusters (Figures 1 and 2). For example, 300, 1,193-3,073, and 556-614 IIGs were found in C-I, C-II, and C-III, respectively. The numbers of IIGs in NILs containing different alleles at a same gene locus also varied (Figures 1 and 2).

**Figure 3:**
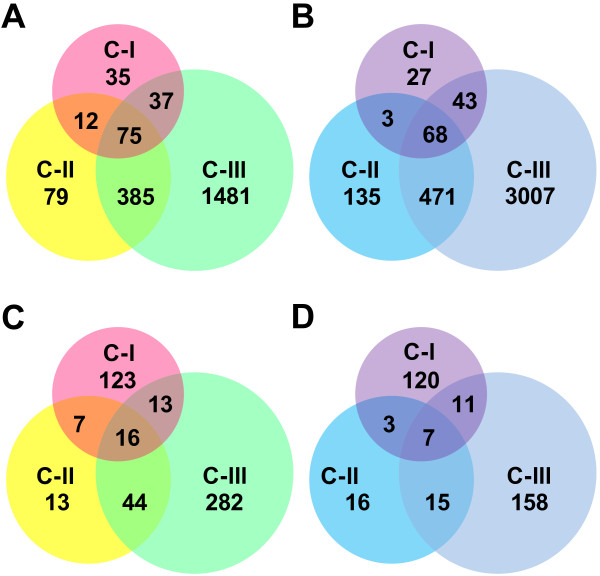
**Comparison of incompatible interaction genes between three clusters, C-I, C-II, and C-III. A**: Broad range comparison for up-regulated genes. **B**: Broad range comparison for down-regulated genes. **C**: Narrow range comparison for up-regulated genes. **D**: Narrow range comparison for down-regulated genes.

The IIGs among the three clusters were analyzed by both broad range comparison, where all the genes within a cluster were counted, and narrow range comparison, where only those genes shared by the NILs within a cluster were counted. Broad range comparison showed that 75 up-regulated and 68 down-regulated IIGs were shared by the three clusters, whereas 62, 214, and 4,488 IIGs were unique to NILs in Clusters I, II, and III, respectively (Figure 3A,B). In contrast, narrow range comparison indicated that only 16 up-regulated and 7 down-regulated IIGs were shared among the three clusters (Figure 3C,D). The annotation of these 23 IIGs is listed in Table 3.

**Table 3:** Incompatible interaction genes (IIGs) shared by all the near isogenic lines and their annotation

**Gene**	**Arabidopsis homologous gene**	**Gene symbol**	**Gene annotation**	**GO group**
**Up regulated**				
Glyma01g32130	AT2G40435	-	Transcription factor, bHLH	Response to biotic or abiotic stress
Glyma02g16710	AT1G03220	-	Eukaryotic aspartyl protease family protein	Metabolism
Glyma03g01010	AT4G26010	-	Peroxidase superfamily protein	Response to biotic or abiotic stress
Glyma05g34870	AT1G14870	PCR2	Plant cadmium resistance 2	-
Glyma06g09220	AT5G25880	NADP-ME3	NADP-malic enzyme 3	Response to biotic or abiotic stress
Glyma06g13090	AT5G24110	WRKY30	WRKY DNA-binding protein 30	Response to biotic or abiotic stress
Glyma09g15090	AT4G21380	RK3	Receptor kinase 3	Metabolism
Glyma11g07430	AT5G03260	LAC11	Laccase 11	Metabolism
Glyma13g17800	AT4G04450	WRKY42	WRKY family transcription factor	Biological regulation
Glyma13g30770	AT1G28480	GRX480	Thioredoxin superfamily protein	Response to biotic or abiotic stress
Glyma13g36070	AT3G20660	4-Oct	Organic cation/carnitine transporter4	Biological regulation
Glyma14g39300	AT1G66160	CMPG1	CYS, MET, PRO, and GLY protein 1	Response to biotic or abiotic stress
Glyma15g05810	AT2G41480	-	Peroxidase superfamily protein	Response to biotic or abiotic stress
Glyma15g06010	AT5G64260	EXL2	Exordium like 2	-
Glyma15g19600	AT2G05940	-	Protein kinase superfamily protein	Metabolism
Glyma20g38000	AT1G09090	RBOHB	Respiratory burst oxidase homolog B	Response to biotic or abiotic stress
**Down regulated**				
Glyma04g12290	AT1G71380	CEL3	Cellulase 3	Response to biotic or abiotic stress
Glyma09g37290	AT5G15230	GASA4	GAST1 protein homolog 4	Response to biotic or abiotic stress
Glyma12g10670	AT3G21700	SGP2	Ras-related small GTP-binding family protein	Response to biotic or abiotic stress
Glyma12g29980	AT4G39370	-	Ubiquitin-specific protease 27	-
Glyma13g44210	AT5G20190	-	Tetratricopeptide repeat (TPR)-like superfamily protein	-
Glyma15g18440	AT4G29720	PAO5	Polyamine oxidase 5	Metabolism
Glyma18g49400	AT5G15230	GASA4	GAST1 protein homolog 4	Response to biotic or abiotic stress

### Putative functions of DEGs based on gene ontology (GO) analysis

**Figure 4:**
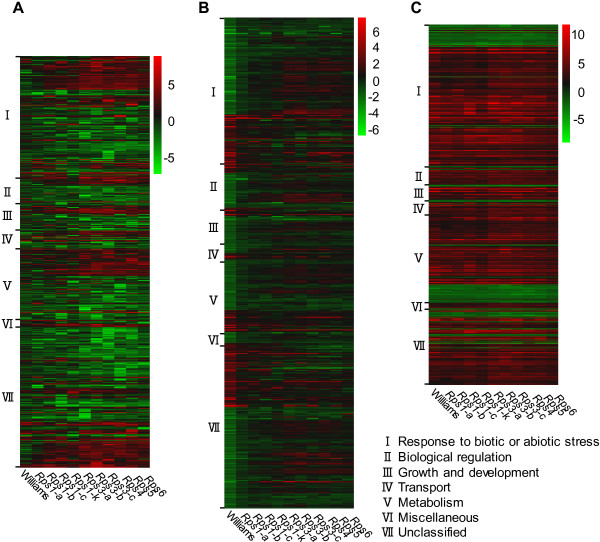
**Heatmap analysis of three categories of differentially expressed genes based on gene ontology analysis.** The values used to draw heatmaps are Log_2_ (fold change) of expression level of post inoculation to mock inoculation. **A**: Incompatible interaction genes. The Log_2_ (fold change) less than 3 in all 10 resistant near isogenic lines were not shown here. **B**: Compatible interaction genes. **C**: Core basal interaction genes.

To understand the functional components involved in molecular responses to *P. sojae*, we annotated the putative products encoded by IIGs, CIGs, and BIGs based on GO analysis (Figure 4; Additional file 6). These DEGs were grouped into seven categories: 1) Response to biotic or abiotic stress; 2) Biological regulation; 3) Growth and development; 4) Transport; 5) Metabolism; 6) Miscellaneous; and 7) Unclassified. For IIGs, the majority of annotated genes fell into the category of “response to biotic stress and abiotc stress” (Figure 4; Additional files 6 and 7). This pattern was also observed for CIGs and BIGs (Figure 4; Additional file 7). Of the 23 IIGs shared by the 10 NILs, 12, 2, and 5, were found to be related to “response to biotic or abiotic stress”, “biological regulation”, and “metabolism”, respectively (Table 3). The remaining four shared genes could not be classified into any functional category.

### Putative transcription factors (TFs) involved in molecular responses to *P. sojae*

It is documented that TFs play important roles in plant defense and stress responses. As such, we annotated and analyzed putative TFs in the sets of DEGs detected in this study based on a soybean TF database that is composed of 5,671 predicted TFs [37]. Of the 5,806 non-redundant IIGs, 282 up-regulated and 543 down-regulated genes were annotated as putative TFs. The up-regulated and down-regulated IIG TFs range from 13 to 177 and from 21 to 307, respectively, among the 10 NILs (Additional files 8 and 9). The number of IIG TFs also varied among the three clusters, with 34, 164, and 791 in C-I, C-II, and C-III, respectively. The most abundant ones along these IIG TFs (either up-regulated or down-regulated) were of the WRKY family, accounting for 23.1% of all IIG TFs identified in C-I, 18.5-25.8% in C-II, and 14.0-18.6% in C-III (Additional file 9). Only three up-regulated IIG TFs (1 bHLH and 2 WRKY) and one down-regulated TF (TPR) were found to be shared by all the 10 NILs (Table 3; Additional file 9).

Of the 1,139 CIGs identified in Williams, 43 up-regulated and 149 down-regulated genes were found to be putative TFs (Additional file 9). The largest proportion of up-regulated CIG TFs belonged to the MYB/HD-like family (16.3%), followed by the AP2-EREBP family (14.0%). In contrast, members of the AP2-EREBP family represented the largest number (20.8%) of down-regulated CIG TFs, followed by MYB/HD-like (10.1%) and bHLH (9.4%). None of these 43 up-regulated CIG TFs belonged to WRKY family.

Of the 835 non-redundant BIGs shared by all the 11 soybean lines, 41 up-regulated and 9 down-regulated ones were found to be TFs (Additional file 9). Among the up-regulated BIG TFs, the most abundant ones are of the WRKY family (34%), followed by the MYB/HD-like family (19.5%).

### Knowledge-based comparative analysis of *Rps* gene-mediated defense signaling pathways

**Figure 5:**
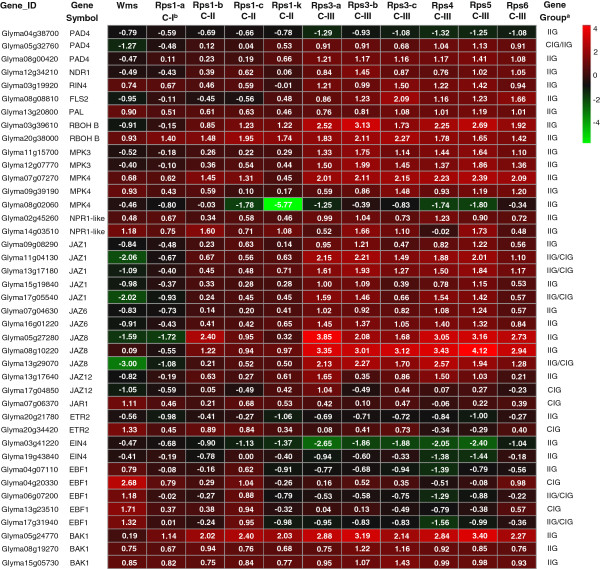
**Expression of selected IIGs or CIGs involved in defense response and defense related phytohormone signaling pathways.** The values and the corresponding depth of different colors in each square indicate Log_2_ (fold change) of expression level of post inoculation to mock inoculation. ^a^Classification of differentially expressed genes. IIG = Incompatible interaction gene. CIG = Compatible interaction gene. ^b^Clustering of *Rps* genes based on hierarchical cluster analysis of IIGs. C-I = Cluster I. C-II = Cluster II. C-III = Cluster III.

The DEGs showing homologies to previously identified protein genes responsible for pathogen recognition and defense, or defense-related phytohormone signaling genes were further analyzed. Overall, these DEGs exhibited diverse patterns of distribution among the three NIL clusters (Figure 5). For example, of the 26 up-regulated IIGs homologous to previously reported defense/stress signaling genes, 24 were found only in NILs within Cluster III. In contrast, only one (*Glyma20g38000*) of the two *RBOH B* gene homologs was found to be up-regulated in all 10 NILs. It is notable that the majority of the putative DEGs involved in defense-related phytohormone signaling pathways showed distinct or opposite patterns of variation in gene expression between Williams and NILs, particularly the NILs within the Cluster III (Figure 5).

Previous studies indicated that the resistance to *P. sojae* in soybean was mediated by SA signaling [24-26], with the *NPR1* as the key component of SA-mediated signaling [38]. In this study, two *NPR1*-like IIGs (*Glyma02g45260* and *Glyma14g03510*) were identified (Figure 5). However, these two genes differ from the two *NPR1*-like genes reported by Sandhu et al [25]. In the later study, they studied soybean leaves four dpi with *P. sojae* race 4 while, in the present study, we studied stems 24 hpi with *P. sojae* race 1. As such, these two studies are less comparable.

As an antagonist of SA, the JA pathway is repressed by JAZ proteins in *Arabidopsis*[39,40]. Several *JAZ* homologs were identified as IIG and/or CIG, including homologs of *JAZ1*, *JAZ6*, *JAZ8* and *JAZ12* (Figure 5). These homologs showed a consistent expression pattern that was up-regulated NILs and down-regulated in Williams. In contrast to the JAZ proteins, *JAR1* (Jasmonate resistant1) appears to be required for the activation of the JA signaling pathway in *Arabidopsis*[41,42]. We found that a soybean *JAR1* homolog (*Glyma07g06370*) was up-regulated in Williams upon inoculation with the *P. sojae*. These observations suggest that the JA signaling pathway may have played opposite roles in phytohormone signaling between incompatible and compatible interactions.

The ET signaling pathway is generally considered to work cooperatively with JA in response to necrotrophic pathogens [8,22]. In *Arabidopsis*, ET receptors *ETR2*[43] and *EIN4*[44] were both negative regulators of ET signaling, and an ubiquitin/proteasome pathway for the degradation of *EIN3* was mediated by an F-box protein, *EBF1*, to negatively regulate ET responses [23]. We found that one homolog of *ETR2* and two homologs of *EIN4* were down-regulated in NILs, while another homolog of *ETR2* was up-regulated in Williams. In addition to *ETR2* and *EIN4*, we identified five *EBF1* homologs that were down-regulated in all NILs and up-regulated in Williams (Figure 5), suggesting that the ET signaling response to the pathogen was repressed during compatible interactions but activated during incompatible interactions.

In addition to the three major phytohormones described above, BR have also been found to enhance disease resistance against viral and bacterial pathogens in tobacco and rice [45]. *BAK1* is a crucial component of BR signaling, and can interact with LRR-RLK (leucine-rich repeat receptor-like protein kinase) genes such as *FLS2* to promote their function in plant defense responses [46]. As shown in Figure 5, three *BAK1* homologs were identified as IIGs and all were up-regulated upon inoculation with the pathogen, suggesting a positive role of BR signaling in defense response to *P. sojae*.

## Discussion

### The nature of distinction in molecular immune response among NILs

One of the most striking observations in this study was the distinction in defense response to inoculation with *P. sojae* among the ten resistant NILs as revealed by comparative transcriptomic analysis. This was reflected not only by the level of variation in the number of IIGs (ranging from 300 to 3073) detected in individual NILs, but also by the relative small numbers of up-regulated (16) and down-regulated (7) IIGs shared by all NILs (Table 3). In general, different *Rps* genes recognize distinct *Avr* genes in a *P. sojae* population [47], but the *Avr* determinants of different *Rps* genes in a same isolate of the pathogen (i.e., race 1 in this study) may not be distinct. One hypothesis that may explain the observed variations of DEGs among different NILs may be differential timing and robustness in defense responses and signaling mediated by different *Rps* genes/alleles [48]. Although the timing of gene expression mediated by different *R* genes in response to the same pathogen has not been characterized in plants, dramatic and rapid changes in gene expression after inoculation with, or following infection by a pathogen, have been observed [24,49]. A recent study demonstrated that ~5% of DEGs were shared by soybean lines resistant to bacterial leaf pustule at 6 and 12 hpi with *Xanthomonas axonopodis*[33]. In this study, the majority (>95%) of DEGs showed less than an eight-fold difference in expression levels upon inoculation with the pathogen when given the same cutoff time for each of the 11 lines. Such a low level of expressional changes and uniformed cutoff may have affected the detectability of shared DEGs among different lines. In addition, the NILs remain 1-2% genomic difference (mostly surrounding the *Rps* loci) from each other, which may have affected expression patterns of a small proportion of genes. Furthermore, inoculated regions of seedling stems, near which layers of cells respond to the pathogen, vary in size among different plants and NILs. Such a variation may affect effective identification of DEGs. Therefore, the numbers of DEGs, and particularly IIGs specific to each NIL could be over-estimated. Nevertheless, the DEGs in each line were detected pre-and post-inoculation with the same pathogen, and thus the influence of genomic difference surrounding *Rps* genes on our pipeline for detection of DEGs triggered by the pathogen may be minimal.

It is documented that resistance genes recognize pathogens either directly or indirectly by guarding a protein or using a decoy [13]. In *Arabidopsis*, *RIN4* is ‘guarded’ by resistance genes *RPM1* and *RPS2*, which trigger the immune response [14,15]. In soybean, two of four *RIN4*- homologous genes appear to function as a heteromeric complex in mediating RPG1-B and RPM1-derived resistance to *Pseudomonas syringae*, and silencing *GmRIN4a* or *GmRIN4b* in *rpg1-b* plants enhances basal resistance to virulent strains of *P. syringae* and the oomycete *P. sojae*[50]. In our study, a single *RIN4* homolog was found to be up-regulated in C-III NILs, but not in NILs within C-I and C-II. If this *RIN4* homolog did encode the “guard” protein that was recognized by the *Rps* genes in the C-III NILs, then the relatively low numbers of IIGs identified among NILs in C-I and C-II, as compared with the C-III NILs, may be explained by the distinct “recognition” processes needed for triggering the immune response, which may result in variations in speed, timing, and magnitude of molecular responses to the pathogen [48,51].

### Do patterns in transcriptomics distinguish *Rps* genes from alleles?

An allele is defined as an alternative form of the same gene that is located at a specific position on a specific chromosome. In general, different alleles of the same gene are determined by genetic tests for allelism. However, due to the complexity of phenotypes, such as variation in the proportion of surviving plants of a pure NIL that contains an *Rps* gene, as observed in this study, such a test may not be effective in determining whether the resistance carried by two individual lines is controlled by different alleles or different genes. Moreover, many NBS-LRR gene models predicted in the Williams82 genome are clustered in a small number of genomic regions [32,52], which makes it more difficult to distinguish genes resistant to the same pathogen by classic genetic analysis, especially if they are physically adjacent or closely linked to each other.

The *Rps* genes/alleles in the 10 NILs have been previously mapped to three genomic regions: *Rps1-a*, *Rps1-b*, *Rps1-c*, and *Rps1-k* on chromosome 3 [6,53,54], *Rps3-a*, *Rps3-b* and *Rps3-c* on chromosome 13 [1,54,55] and *Rps4*, *Rps5* and *Rps*6 on chromosome 18 [1,54,55]. However, because limited numbers of molecular markers were available and used when these genes/alleles were identified, the genetic distances between genes or alleles mapped in the same chromosomal regions have not been determined. Actually, the alleles at the *Rps1* and *Rps3* loci were simply designated based on their disease reaction to a set of *P. sojae* races. As these three regions are highly enriched with NBS-LRR genes, according to the soybean reference genome, it is possible that some designated alleles of the same gene may be located at different loci.

As different alleles of the same gene are generally more identical to each other than to other genes, people are inclined to speculate that the defense response mediated by different alleles of a same gene may be more similar than those mediated by different genes. At first glance, this hypothesis seems to be supported by the observation that the NILs with *Rps3-a* and *Rps3-b* exhibited similar patterns of transcriptional changes upon inoculation with *P. sojae*, as did the NILs with *Rps1-b* and *Rps1-c* (Figure 2). However, the pattern of transcriptional changes mediated by *Rps3-c* was found to be most similar to those mediated by *Rps4*, *Rps5*, and *Rps6*. Thus, the patterns of transcriptomic changes do not appear to predict genetic similarity and difference among these *Rps* genes and alleles, although we could not rule out the possibility that *Rps3-c* and *Rps1-a* are non-allelic to the *Rps3* and *Rps1* loci, respectively.

### An integrated picture of molecular responses to *P. sojae*

**Figure 6:**
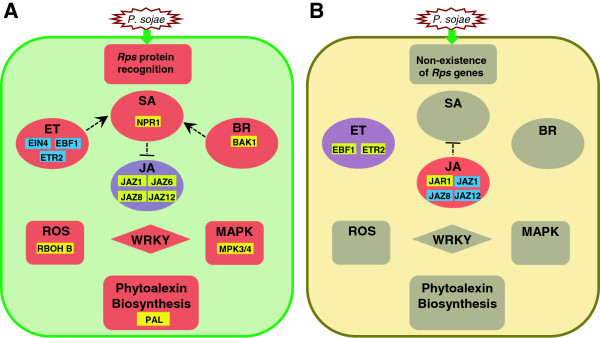
**An integrated picture for gene network of interactions between soybean and race1 of *Phytophthora sojae. *A**: Incompatible interaction. **B**: Compatible interaction. Red = up-regulation of a pathway. Purple = down-regulation of a pathway. Grey = no significant change in pathway. Yellow = up-regulation of genes. Blue = down-regulation of genes. Dotted lines indicate interactions of pathways inferred from *Arabidopsis* studies.

Based on analysis of putative defense-related genes identified in this study and the patterns of their expressions upon inoculation with the pathogen, we propose a hypothesis that four major phytohormone signaling pathways are involved in defense responses (Figure 6A). During incompatible interaction, the *Rps* protein recognizes the avirulence effector of *P. sojae*, initiating signaling transduction that involves the SA, JA, ET and BR pathways. The SA, ET, and BR pathways are activated, whereas the JA pathway is suppressed. Although the specific functions of individual genes encoding TFs belonging to the WRKY family, as well as those genes for reactive oxygen species (ROS) and MAP kinase (MAPK) cascades remain to be elucidated, it is apparent that some of the genes belonging to these families play pivotal roles in defense signaling transduction. During compatible interaction, the avirulence effector of *P. sojae* induces a set of downstream responses that lead to disease development (Figure 6B). The signatures of the susceptible responses would include up-regulation of the JA pathway, suppression of ET pathway and with no significant changes in SA and BR pathways. It seems possible that the defense responses might be delayed in the compatible interaction due to insufficient activation of the expression of genes related to ROS and phytoalexin biosynthesis, as well as MAPK signaling. Together, these data gained from a unique set of soybean NILs provide a comprehensive picture regarding the molecular mechanisms underlying incompatible and compatible interaction between soybean and *P. sojae*, which shed light on the nature and timing of molecular responses mediated by individual *Rps* genes.

## Conclusions

Comparative transcriptomic analysis of ten resistant NILs and susceptible recurrent line Williams with and without inoculation allowed us to identify DEGs associated with defense responses to *P. sojae* that were unique in each NIL and common among all these NILs, and thus depicted functional “fingerprints” of individual *Rps*-mediated resistance. Further analysis revealed multiple branches of putative ET, JA, ROS, and MAPK regulatory networks underlying the defense responses. Such responses exhibited dramatic variations among the soybean NILs containing distinct *Rps* genes/alleles. We propose different timing of individual *Rps*-mediated defense signaling to the same pathogen may be largely responsible for such variations.

## Methods

### Plant materials and isolates of *P. sojae*

The susceptible cultivar Williams and its ten NILs, each containing a unique *Rps* gene/allele, were used this study (Additional file 1). Each NIL was generated by backcrossing the donor for at least five generations with Williams. For inoculation tests, the isolate *pmg(1)-3* (pathotype race1) of *P. sojae* was used.

### Treatment of soybean materials

Seeds of each soybean line were planted in sterilized sand in 10.1 cm clay pots and placed in a greenhouse. Approximately seven days after planting, at the VC growth stage (unifoliate leave fully expanded), the seedlings for each line were separated into four groups, each containing ~20 seedlings. Two groups were challenged with *P. sojae* using a standard hypototyl inoculation method [56], while the other two groups were mock inoculated (wounded) without the pathogen. At 24 hpi, stems were harvested from one set of inoculated and one set of wounded seedlings by excising 2 to 3 cm across the wounded site and storing immediately in liquid nitrogen. The seedlings in the remaining two groups were kept for evaluating symptom development, which was recorded 7 dpi. Experiments were performed a total of three times.

### RNA sequencing and analysis

Total RNAs were isolated using the RNeasy Plant Mini Kit (Qiagen Inc., Valencia, CA) according to the manufacturer’s instructions in conjunction with DNase. The quality of total RNA was determined using a Nanodrop spectrometer (Thermo Fisher Scientific Inc., Wilmington, DE) and 1% formaldehyde gel electrophoresis. Total RNA samples were then sent to the Genomics Center at Purdue University for sequencing and 101 bp paired-end reads were generated with the Illumina HiScanSQ system. Since samples were a mixture of RNA from both soybean and *P. sojae*, to exclude the effect of *P. sojae* sequences, all reads that aligned to the *P. sojae* genome sequence were eliminated [57]. All remaining reads were then aligned to *G. max* reference genome (v8.0, http://www.phytozome.net) using TopHat software [58] with parameters set to allow only one mismatch, and 30 and 100,000 bp of the minimum and maximum intron length based on the current gene annotation. Moreover, only the uniquely mapped reads or fragments were kept for further analyses. The raw count of each gene was calculated by HTSeq (http://www-huber.embl.de/users/anders/HTSeq) with the “intersection_nonempty” mode, and preceded to edgeR package [59] in the R-Bioconductor tools.

To detect variability, we estimated the over dispersion from 40 highly confident soybean housekeeping genes collected from previously reported papers (Additional file 10) [24,33,60-63], considering different soybean lines as replicates of control and inoculation groups. The data were then modeled using a Negative Binomial model in edgeR to identify differentially expressed genes after inoculation using the dispersion estimated from the housekeeping genes as a common dispersion. Any genes with coverage read count less than one count per million of two paired samples were removed in later analyses. Differential expression between the inoculation group and the mock-inoculation group for each line was tested for false discovery rate (FDR) that was controlled at 0.05 [64] using edgeR package [59].

### Hierarchical cluster and heatmap analysis

Pvclust and hclust were performed using the log_2_FC of the 5,806 IIGs with default parameters using distance method “correlation” for complete linkage clustering analysis. Pvclust provides two types of *p*-values to assess the uncertainty for each cluster: approximately unbiased (AU) p-value and bootstrap probability (BP) value, via multi-scale bootstrap re-sampling [35]. The heatmap representing the expression intensity and direction was drawn using pheatmap *R* package with the distance method “euclidean” for both rows and columns [65].

### Gene Ontology, heatmap and homologous gene functional analysis

Annotations of GO terms were obtained from the AgriGO website based on the *G. max* model [66], and GO biological process categories were used. These terms were manually classified into six broad functional groups, ‘response to biotic or abiotic stress’, ‘biological regulation’, ‘growth and development’, ‘transport’, ‘metabolism’, and ‘miscellaneous’, while genes without GO annotations were grouped as ‘unclassified’. For genes with multiple GO categories, only one category was selected based on priority. Homologous genes were searched against annotated *Arabidopsis* gene protein database (The *Arabidopsis* Information Resouces, http://www.arabidopsis.org) using BLASTP to verify gene functions manually.

### Quantitative real-time PCR analysis

The qRT-PCR was carried out using StepOnePlus™ Real Time PCR system (Applied Biosystems). The RNA samples used as templates for qRT-PCR were the same as those used for RNA-Seq. The *cons4* gene [60] was used as internal control for normalization of qRT-PCR data. For each gene, three experimental replicates were performed. Pearson correlations were calculated between RNA-Seq and qRT-PCR methods using six out of seven randomly selected genes for Log2 fold change of inoculated and mock-inoculated treatments in each soybean line (Additional file 3).

### Availability of supporting data

The transcriptome sequences presented in this study have been deposited in NCBI Gene Expression Omnibus under accession numbers GSE48524.

## Competing interests

The authors declare that they have no competing interests.

## Authors’ contributions

TJH and JM designed the research; FL, MZ, RWD, TJH, and JM interpreted the data; FL, MZ, DDB, JP, LS, YL, BZ, ZT performed the research; FL, MZ, RWD, TJH, and JM drafted and revised the manuscript; FL, MZ, DDB, JP, LS, YL, BZ, ZT, RWD, TJH, and JM approved the submission of the version. All authors read and approved the final manuscript.

## Supplementary Material

Additional file 1 Breeding pedigree of soybean NILs used in this study. Click here for file

Additional file 2 **Reactions of soybean NILs to *Phytophthora sojae*.** Click here for file

Additional file 3 List of primers used in qPCR. Click here for file

Additional file 4 **Comparison in expression of six soybean genes in Williams and 10 NILs, each containing a single *Rps* gene, as determined by RNA-Seq analysis or qRT-PCR.** Y axis indicates differential expression of selected genes for each soybean line. X axis indicates selected genes used for qRT-PCR. These genes are Glyma02g47940 (1), Glyma04g20330 (2), Glyma05g24770 (3), Glyma07g07270 (4), Glyma09g37290 (5), Glyma10g44170 (6), and Glyma11g04130 (7). Pearson’s corrleation coefficient (r). Click here for file

Additional file 5 **Heatmap (hcluster) analysis of 5,806 IIGs for 10 soybean NILs, each containing a single *Rps* gene.** The values used to draw heatmap is Log_2_ (fold change) of expression level of post inoculation to mock inoculation. Click here for file

Additional file 6 GO analysis of DEGs in different categories. Click here for file

Additional file 7 **Gene ontology categories for IIGs identified in soybean NILs, each containing a single *Rps* gene.** Left bar = up-regulated genes. Right bar = down-regulated genes. Click here for file

Additional file 8 **Comparison of differentially expressed transcription factors (DETFs) between Williams and 10 NILs each containing a single *Rps* gene.** A: up-regulated DETFs. B: down-regulated DETFs. Red and purple represent the number of DETFs specific to individual *Rps* genes and collectively referred to as incompatible interaction transcription factors. Brown and dark purple represent the number of DETFs shared by an individual NIL and Williams. Green and light blue represent the number of DETFs specifically expressed in Williams when compared to a specific NIL. The central green and light blue circles represent the common proportion of Williams-specific DETFs that are collectively referred to as compatible interaction transcription factors. Click here for file

Additional file 9 Counts of transcription factors (TFs) in different groups. Click here for file

Additional file 10 Housekeeping genes selected in this study. Click here for file
