# Supplementary material for: Molecular response to the pathogen Phytophthora sojae among ten soybean near isogenic lines revealed by comparative transcriptomics
Source: BMC Genomics. 2014 Jan 10;15:18. doi: 10.1186/1471-2164-15-18 (PMC3893405; doi:10.1186/1471-2164-15-18)
Supplement: Additional file 1 — Breeding pedigree of soybean NILs used in this study. [file 1471-2164-15-18-S1.docx]

| **Additional file 1** Breeding pedigree of soybean NILs used in this study | | | |
| --- | --- | --- | --- |
| **NIL** | ***Rps* gene** | **Pedigree** | **Donor Origin** |
| Union | *1-a* | Williams(5) x SL12^a^ | - |
| L77-1863 | *1-b* | Williams (7) x Harrel | Virginia, United States |
| L75-3735 | *1-c* | Williams (6) x Lee68 | Arkansas, United States |
| L77-1794 | *1-k* | Williams (7) x Kingwa | Beijing, China |
| L83-570 | *3-a* | Williams (6) x PI 86972-1 | Cholla Puk, Korea, South |
| L91-8347 | *3-b* | Williams (6) x PI 172901 | Artvin, Turkey |
| L92-7857 | *3-c* | Williams (6) x PI 340046 | Kyongsang Puk, Korea, South |
| L85-2352 | *4* | Williams (6) x PI 86050 | Unverified/Hokkaido, Japan |
| L85-3059 | *5* | Williams (6) x PI 91160 | Liaoning, China |
| L89-1581 | *6* | Williams (6) x Altona | Manitoba, Canada |
| ^a^SL12 from [(Wayne(6) x Clark 63) x (Wayne(4) x L11)] x SL9; L11 is from (Clark(6) x T201) x (Clark(6) x T145); SL9 is from Wayne(10) x Kanrich | | | |
