# Supplementary material for: Molecular response to the pathogen Phytophthora sojae among ten soybean near isogenic lines revealed by comparative transcriptomics
Source: BMC Genomics. 2014 Jan 10;15:18. doi: 10.1186/1471-2164-15-18 (PMC3893405; doi:10.1186/1471-2164-15-18)
Supplement: Additional file 2 — Reactions of soybean NILs to Phytophthora sojae . [file 1471-2164-15-18-S2.docx]

| **Additional file 2** Reactions of soybean NILs to *Phytophthora sojae* | | | | | | | | | | | | | | | |
| --- | --- | --- | --- | --- | --- | --- | --- | --- | --- | --- | --- | --- | --- | --- | --- |
|  |  | **Experimental Rep 1** | | | |  | **Experimental Rep 2^a^** | | | |  | **Experimental Rep 3** | | | |
|  |  | **Inoculated** | | **Mock^b^** | |  | **Inoculated** | | **Mock** | |  | **Inoculated** | | **Mock** | |
| **NIL** | ***Rps*** | **#^c^** | **%^d^** | **#** | **%** |  | **#** | **%** | **#** | **%** |  | **#** | **%** | **#** | **%** |
| Williams | rps | 17 | 6 | 13 | 100 |  | 18 | 0 | 20 | 100 |  | 20 | 0 | 20 | 100 |
| Union | 1-a | 16 | 100 | 18 | 100 |  | 20 | 100 | 0^e^ | - |  | 19 | 100 | 19 | 100 |
| L77-1863 | 1-b | 14 | 100 | 15 | 100 |  | 20 | 100 | 0 | - |  | 21 | 100 | 21 | 100 |
| L75-3735 | 1-c | 16 | 100 | 12 | 100 |  | 19 | 100 | 21 | 100 |  | 22 | 100 | 18 | 100 |
| Williams82 | 1-k | 14 | 100 | 8 | 100 |  | 21 | 100 | 19 | 100 |  | 22 | 100 | 20 | 100 |
| L83-570 | 3-a | 16 | 75 | 18 | 100 |  | 22 | 100 | 23 | 100 |  | 21 | 100 | 22 | 100 |
| L91-8347 | 3-b | 14 | 100 | 14 | 100 |  | 20 | 100 | 0 | - |  | 21 | 82 | 22 | 100 |
| L92-7857 | 3-c | 10 | 80 | 13 | 100 |  | 15 | 93 | 19 | 100 |  | 21 | 100 | 21 | 100 |
| L85-2352 | 4 | 17 | 100 | 15 | 100 |  | 18 | 100 | 22 | 100 |  | 21 | 100 | 22 | 100 |
| L85-3059 | 5 | 14 | 86 | 10 | 100 |  | 14 | 100 | 21 | 100 |  | 22 | 95 | 22 | 100 |
| L89-1581 | 6 | 15 | 87 | 14 | 100 |  | 20 | 100 | 21 | 100 |  | 20 | 100 | 22 | 100 |
| ^a^Seedlings were bulked for each line for sequencing. | | | | | | | | | | | | | | | |
| ^b^Seedlings treated in the same manner as inoculated but without *P. sojae*. | | | | | | | | | | | | | | | |
| ^c^#=Number of seedlings observed. | | | | | | | | | | | | | | | |
| ^d^%=Percentage of surviving seedlings. | | | | | | | | | | | | | | | |
| ^e^Due to poor seed germination, no seedlings were reserved for symptom evaluation | | | | | | | | | | | | | | | |
