# Supplementary material for: Molecular response to the pathogen Phytophthora sojae among ten soybean near isogenic lines revealed by comparative transcriptomics
Source: BMC Genomics. 2014 Jan 10;15:18. doi: 10.1186/1471-2164-15-18 (PMC3893405; doi:10.1186/1471-2164-15-18)
Supplement: Additional file 3 — List of primers used in qPCR. [file 1471-2164-15-18-S3.docx]

| **Additional file 3** List of primers used in qPCR | | |
| --- | --- | --- |
| **Gene_ID** | **Forward primer** | **Reverse primer** |
| Glyma02g47940 | TTGGGATGGGAGACCCTTTC | CAAGTTTGTGAGGGAGGAGTTAGAG |
| Glyma04g20330 | CATTAGTTGCCTTCTTCCCTTC | GCGGTGGCTTATGCTCATG |
| Glyma05g24770 | TTCAAGTGGTGGTTGTGATTCC | GAACTCAGGGTGGGGAGATG |
| Glyma07g07270 | AACATAATCTTTGCCAGGAAAGAGT | GTATGTGGTCACTAGATGGTACCGT |
| Glyma09g37290 | GGGGCACACACAGGCATG | CAGTGGTAATGGCTGCTAATGAA |
| Glyma10g44170 | GAAAACCAGTAGAAAGCAGACAATAC | TGCATCAGAGGAGGGTCTACAT |
| Glyma11g04130 | TCTCTCATCATCTTCTGAATAAACATC | TCTGCTAATCCCACTAGACCATTT |
