# Supplementary material for: Molecular response to the pathogen Phytophthora sojae among ten soybean near isogenic lines revealed by comparative transcriptomics
Source: BMC Genomics. 2014 Jan 10;15:18. doi: 10.1186/1471-2164-15-18 (PMC3893405; doi:10.1186/1471-2164-15-18)
Supplement: Additional file 4 — Comparison in expression of six soybean genes in Williams and 10 NILs, each containing a single Rps gene, as determined by RNA-Seq analysis or qRT-PCR. Y axis indicates differential expression of selected genes for each soybean line. X axis indicates selected genes used for qRT-PCR. These genes are Glyma02g47940 (1), Glyma04g20330 (2), Glyma05g24770 (3), Glyma07g07270 (4), Glyma09g37290 (5), Glyma10g44170 (6), and Glyma11g04130 (7). Pearson’s corrleation coefficient (r). [file 1471-2164-15-18-S4.pdf]

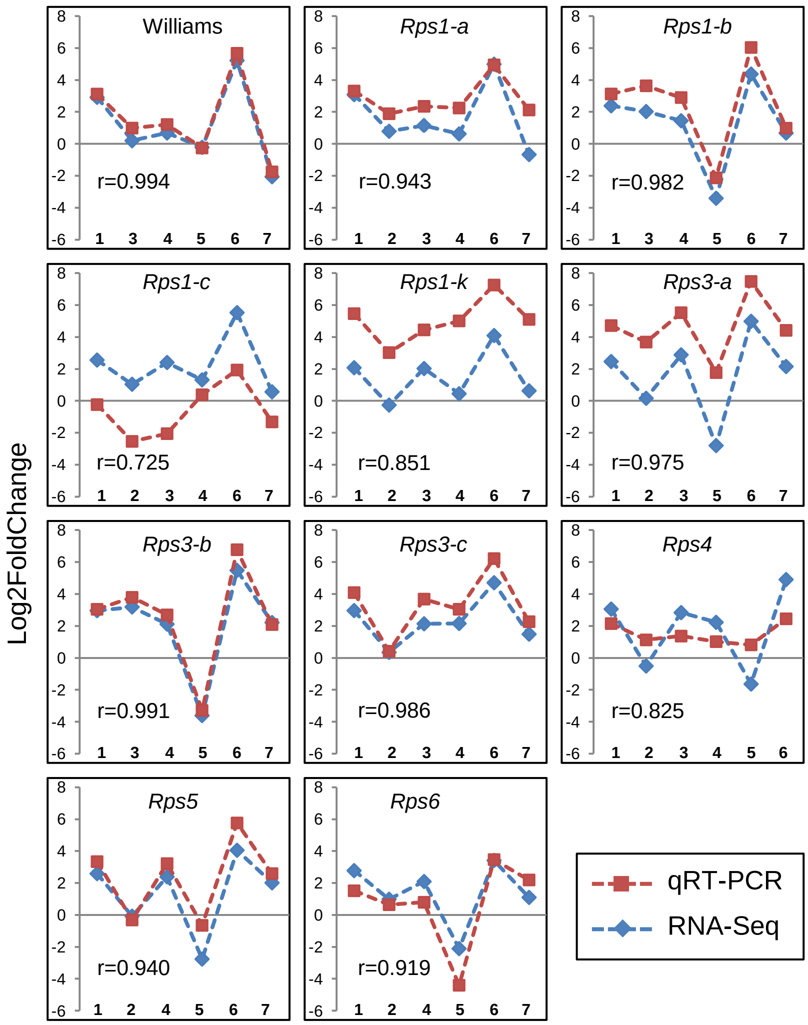

Additional file 4 Comparison in expression of six soybean genes in Williams and 10 NILs, each containing a single *Rps* gene, as determined by RNA-Seq analysis or qRT-PCR.

Y axis indicates differential expression of selected genes for each soybean line. X axis indicates selected genes used for qRT-PCR. These genes are Glyma02g47940 (1), Glyma04g20330 (2), Glyma05g24770 (3), Glyma07g07270 (4), Glyma09g37290 (5), Glyma10g44170 (6), and Glyma11g04130 (7). Pearson's corrleation coefficient (r).
