# Supplementary material for: Molecular response to the pathogen Phytophthora sojae among ten soybean near isogenic lines revealed by comparative transcriptomics
Source: BMC Genomics. 2014 Jan 10;15:18. doi: 10.1186/1471-2164-15-18 (PMC3893405; doi:10.1186/1471-2164-15-18)
Supplement: Additional file 5 — Heatmap (hcluster) analysis of 5,806 IIGs for 10 soybean NILs, each containing a single Rps gene. The values used to draw heatmap is Log2 (fold change) of expression level of post inoculation to mock inoculation. [file 1471-2164-15-18-S5.pdf]

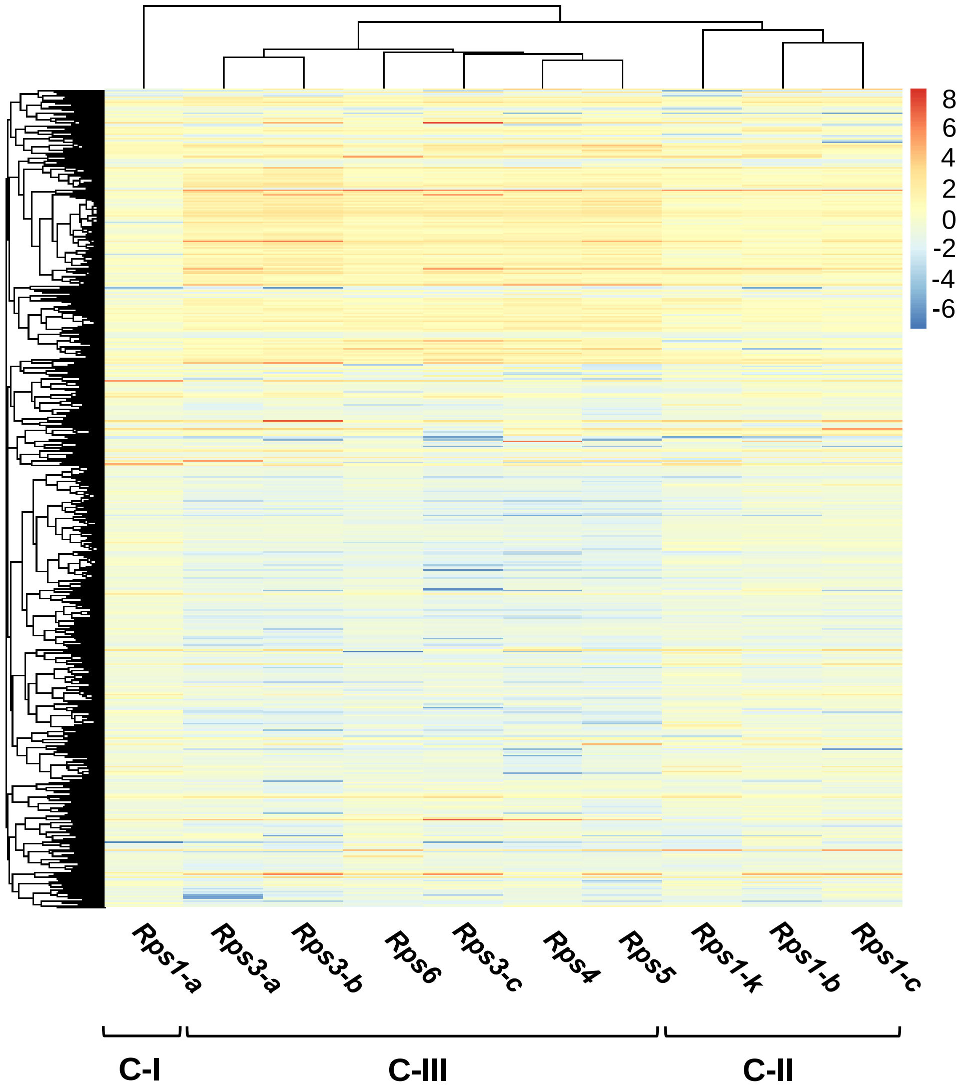

Additional file 5 Heatmap (hcluster) analysis of 5,806 IIGs for 10 soybean NILs, each containing a single *Rps* gene. The values used to draw heatmap is Log_2 (fold change) of expression level of post inoculation to mock inoculation.
