# Supplementary material for: Molecular response to the pathogen Phytophthora sojae among ten soybean near isogenic lines revealed by comparative transcriptomics
Source: BMC Genomics. 2014 Jan 10;15:18. doi: 10.1186/1471-2164-15-18 (PMC3893405; doi:10.1186/1471-2164-15-18)
Supplement: Additional file 7 — Gene ontology categories for IIGs identified in soybean NILs, each containing a single Rps gene. Left bar = up-regulated genes. Right bar = down-regulated genes. [file 1471-2164-15-18-S7.pdf]

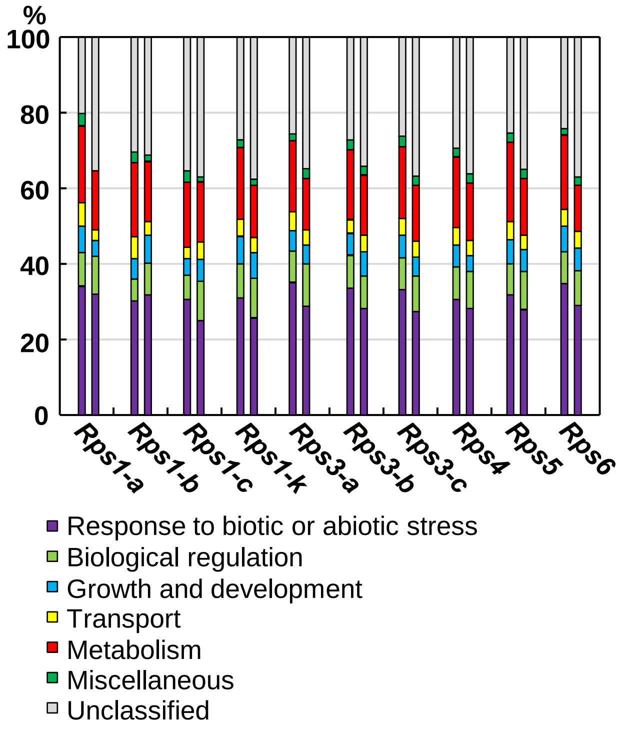

Additional file 7 Gene ontology categories for IIGs identified in soybean NILs, each containing a single *Rps* gene. Left bar = up-regulated genes. Right bar = down-regulated genes.
