# Supplementary material for: Molecular response to the pathogen Phytophthora sojae among ten soybean near isogenic lines revealed by comparative transcriptomics
Source: BMC Genomics. 2014 Jan 10;15:18. doi: 10.1186/1471-2164-15-18 (PMC3893405; doi:10.1186/1471-2164-15-18)
Supplement: Additional file 8 — Comparison of differentially expressed transcription factors (DETFs) between Williams and 10 NILs each containing a single Rps gene. A: up-regulated DETFs. B: down-regulated DETFs. Red and purple represent the number of DETFs specific to individual Rps genes and collectively referred to as incompatible interaction transcription factors. Brown and dark purple represent the number of DETFs shared by an individual NIL and Williams. Green and light blue represent the number of DETFs specifically expressed in Williams when compared to a specific NIL. The central green and light blue circles represent the common proportion of Williams-specific DETFs that are collectively referred to as compatible interaction transcription factors. [file 1471-2164-15-18-S8.pdf]

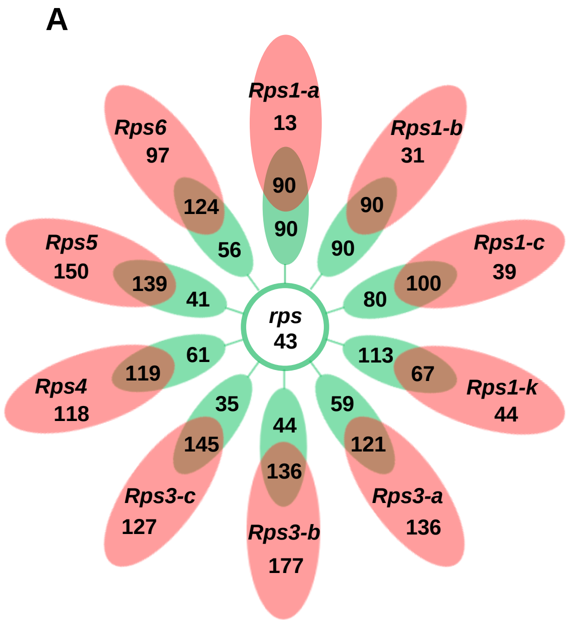

Additional file 8 Comparison of differentially expressed transcription factors (DETFs) between Williams and 10 NILs each containing a single *Rps* gene. A: up-regulated DETFs. B: down-regulated DETFs. Red and purple represent the number of DETFs specific to individual *Rps* genes and collectively referred to as incompatible interaction transcription factors. Brown and dark purple represent the number of DETFs shared by an individual NIL and Williams. Green and light blue represent the number of DETFs specifically expressed in Williams when compared to a specific NIL. The central green and light blue circles represent the common proportion of Williams-specific DETFs that are collectively referred to as compatible interaction transcription factors.
