# Supplementary material for: Molecular response to the pathogen Phytophthora sojae among ten soybean near isogenic lines revealed by comparative transcriptomics
Source: BMC Genomics. 2014 Jan 10;15:18. doi: 10.1186/1471-2164-15-18 (PMC3893405; doi:10.1186/1471-2164-15-18)
Supplement: Additional file 10 — Housekeeping genes selected in this study. [file 1471-2164-15-18-S10.docx]

**Additional file 10** Housekeeping genes selected in this study

| **Gene ID** | **Gene symbol** | **NCBI Accession No.** | **Reference** |
| --- | --- | --- | --- |
| Glyma02g44460 | EF1b | EV279336 | Hu et al. 2009; Jian et al. 2008 |
| Glyma03g27970 | TUB4 | EV263740 | Hu et al. 2009 |
| Glyma03g29350 | Cons7/MTP | AW310136/CF808703 | Libault et al. 2008; Hu et al. 2009 |
| Glyma04g02610 | Tubulin_motif | - | Kim et al. 2011 |
| Glyma04g05080 | Cons17 | AI442942 | Libault et al. 2008 |
| Glyma04g39380 | ACT2/7 | BW677100 | Hu et al. 2009; Jian et al. 2008 |
| Glyma05g23230 | Tubulin_motif | - | Kim et al. 2011 |
| Glyma05g29000 | TUA5 | CA801144 | Hu et al. 2009 |
| Glyma05g37860 | Cons10 | CD394504 | Libault et al. 2008 |
| Glyma06g01850 | GmGAPDH | DQ224371.1 | Stolf-Moreira et al. 2011 |
| Glyma06g04180 | UKN2 | BE330043 | Hu et al. 2009 |
| Glyma07g32020 | UBQ10 | EH258122 | Hu et al. 2009; Jian et al. 2008 |
| Glyma08g01740 | Tubulin | - | Kim et al. 2011 |
| Glyma08g05480 | HDC | CK768960 | Hu et al. 2009 |
| Glyma08g19420 | Actin | - | Kim et al. 2011 |
| Glyma10g17160 | Cons8 | BG508491 | Libault et al. 2008 |
| Glyma10g29280 | Cons9 | BI317557 | Libault et al. 2008 |
| Glyma10g38460 | Cons15/PEPKR1 | AW396185 | Hu et al. 2009; Libault et al. 2008 |
| Glyma11g04750 | Tubulin_motif | - | Kim et al. 2011 |
| Glyma12g02310 | Cons4/UKN1 | BU578186 | Hu et al. 2009; Kim et al. 2011; Libault et al. 2008 |
| Glyma12g02790 | CYP | CF806591 | Hu et al. 2009; Jian et al. 2008 |
| Glyma12g05510 | Cons6/SKIP16 | CD397253 | Hu et al. 2009; Kim et al. 2011; Libault et al. 2008 |
| Glyma12g36300 | Cons18 | BG157306 | Libault et al. 2008 |
| Glyma13g12030 | GmRNAr18S | X02623.1 | Stolf-Moreira et al. 2011 |
| Glyma14g07680 | Cons16 | AI938444 | Libault et al. 2008 |
| Glyma14g40380 | Cons3 | BE659234 | Libault et al. 2008 |
| Glyma15g05570 | Gmβ-actin | GMU60500 | Stolf-Moreira et al. 2011 |
| Glyma15g13970 | Tubulin_motif | - | Kim et al. 2011 |
| Glyma15g15010 | Cons5 | CA801096 | Libault et al. 2008 |
| Glyma15g42570 | Cons1 | BU551231 | Libault et al. 2008 |
| Glyma16g32510 | Cons14 | BU548932 | Libault et al. 2008 |
| Glyma17g08010 | Cons11 | CD391856 | Libault et al. 2008 |
| Glyma17g14750 | Cons2 | AW348242 | Libault et al. 2008 |
| Glyma18g16160 | UBC2 | - | Jian et al. 2008 |
| Glyma18g52780 | ACT11 | BW652479 | Hu et al. 2009; Jian et al. 2008 |
| Glyma19g37940 | Tubulin_motif | - | Kim et al. 2011 |
| Glyma20g26690 | TIP41 | EV263725 | Hu et al. 2009 |
| Glyma20g27280 | Tubulin_motif | - | Kim et al. 2011 |
| Glyma20g29840 | Tubulin_motif | - | Kim et al. 2011 |
| Glyma13g17820 | UBQ10 |  | Jian et al. 2008 |
